# Caries Experience among Adults Exposed to Low to Moderate Doses of Ionizing Radiation in Childhood – The Tinea Capitis Cohort

**DOI:** 10.3389/fpubh.2016.00018

**Published:** 2016-02-23

**Authors:** Yuval Vered, Angela Chetrit, Harold D. Sgan-Cohen, Tova Amitai, Jonathan Mann, Hadas Even-Nir, Siegal Sadetzki

**Affiliations:** ^1^Department of Community Dentistry, Hadassah Faculty of Dental Medicine, Hebrew University, Jerusalem, Israel; ^2^Cancer and Radiation Epidemiology Unit, Chaim Sheba Medical Center, Gertner Institute for Epidemiology and Health Policy Research, Tel Hashomer, Israel; ^3^Sackler Faculty of Medicine, Tel Aviv University, Tel Aviv, Israel

**Keywords:** ionizing radiation, dental caries, public health, DMF index, risk assessment

## Abstract

While the impact of therapeutic levels of ionizing radiation during childhood on dental defects has been documented, the possible effect of low doses on dental health is unknown. The study aim was to assess the association between childhood exposure to low–moderate doses of therapeutic radiation and caries experience among a cohort of adults 50 years following the exposure. The analysis was based on a sample of 253 irradiated (in the treatment of tinea capitis) and 162 non-irradiated subjects. The decayed, missing, and filled teeth (DMFT) index was assessed during a clinical dental examination and questions regarding dental care services utilization, oral hygiene behavior, current self-perceived mouth dryness, socio-demographic parameters, and health behavior variables were obtained through a face-to-face interview. An ordered multivariate logistic regression model was used to assess the association of the main independent variable (irradiation status) and other relevant independent variables on the increase in DMFT. Mean caries experience levels (DMFT) were 18.6 ± 7.5 for irradiated subjects compared to 16.4 ± 7.2 for the non-irradiated (*p* = 0.002). Controlling for gender, age, education, income, smoking, dental visit in the last year, and brushing teeth behavior, irradiation was associated with a 72% increased risk for higher DMFT level (95% CI: 1.19–2.50). A quantification of the risk by dose absorbed in the salivary gland and in the thyroid gland showed adjusted ORs of 2.21 per 1 Gy (95% CI: 1.40–3.50) and 1.05 per 1 cGy (95% CI: 1.01–1.09), respectively. Childhood exposure to ionizing radiation (0.2–0.4 Gy) might be associated with late outcomes of dental health. In line with the guidelines of the American Dental Association, these results call for caution when using dental radiographs.

## Introduction

Previous studies have shown that exposure of the head to therapeutic levels of ionizing radiation (IR) are associated with both acute and chronic oral complications (1–3).

Studies have reported that irradiation dosage as low as 2 Gy (Gray) might cause salivary gland dysfunction and xerostomia (4), while permanent damage has been reported at dose levels reaching 20–60 Gy (5). Salivary gland tumors have also been observed following exposure to doses of 0.4 Gy (6) and 0.78 Gy (7). It is well established that any impediment of the physiologic salivary protection may have an essential role in carcinogenesis (8).

Dental defects have been attributed to irradiation during childhood, while teeth are developing. Studies among long-term survivors of childhood cancer (4–60 Gy), demonstrated late dental and maxillofacial effects, and the severity of damage was negatively associated with age at exposure and positively related to dose (9–11).

While the association between IR and cancer is well established (12), the effects of low–moderate doses of radiation on health outcomes other than cancer are still under study (13).

“Between 1946 and 1960, about 20,000 Israeli children were treated with IR to the head for tinea capitis (TC), a benign fungal disease of the scalp” (14–16). “In 1968, a comprehensive follow-up of a cohort including the irradiated group and two comparison groups was initiated to determine possible delayed side effects of irradiation” (16–18). The aim of the present study was to assess the association of childhood exposure to low–moderate doses of IR on dental caries cumulative experience.

## Materials and Methods

The study protocol was approved by the ethics panel of the Chaim Sheba Medical Center (reference number: 3596) and informed consent was obtained from all participants.

The “TC cohort” was comprised of 10,834 Jewish subjects irradiated for TC. “The comparison group included 10,834 non-irradiated subjects derived from the National Population Registry, matched to the exposed subjects by age (±2 years), gender, country of birth, and year of immigration. The TC Study cohort and the irradiation treatment have been described and analyzed in several previous studies” (15–19). “The analysis presented here is based on a random subsample of irradiated and non-irradiated subjects from the TC cohort. The inclusion criteria were residency in two large cities in Israel and being free of malignant disease. Exclusion criteria included death and medical conditions that did not allow an interview such as mental disease. The sample of individuals who comply with these eligibility criteria included 827 individuals (426 irradiated and 401 non-exposed population controls)” (16). Of them, “20% (*n* = 171) could not participate in the study due to unavailable addresses and 415 individuals were interviewed (79 and 48% of the traceable irradiated and non-irradiated groups respectively). Participants and non-participants of each study group did not differ significantly by gender and place of birth, and by age in the irradiated group. Participants of the non-irradiated group tended to be slightly younger than non-participants (mean ages 58 vs. 59 years, respectively; *p* = 0.02)” (16).

“Treatment of TC had involved application of the Adamson-Kienbock technique. Prior to irradiation, the subject’s hair was shaved and remaining hair was removed by a waxing process. In the 1960s, dosimetry was estimated retrospectively using one of the original X-ray machines and a specially designed head phantom” (16). In the 1980s, individual average doses to different organs were estimated for each irradiated case. “These estimations were based on the measurements made on an anthropomorphic phantom, the prescribed medical center-specific exposure technique, the number of treatment courses and age and gender (which were highly correlated with size of the child). The mean average doses for all irradiated individuals were 1.5, 0.09, and 0.78 Gy to the brain, thyroid and parotid glands respectively (7). The estimated dose to the teeth ranged between 0.2 to 0.4 Gy, with the higher dose to the posterior molars” (16).

Data were gathered in a personal meeting comprising an interview and a dental examination that was performed by one senior dentist (Yuval Vered). The questionnaire included data about socio-demographic parameters, health behavior variables, history of diseases and hospitalizations, and past exposure to irradiation. This questionnaire had been used in several previous studies of the TC cohort (20).

The dental abstraction form included questions regarding dental care services utilization, oral hygiene behavior, and current self-perceived mouth dryness.

The results of the clinical dental examination were recorded employing the decayed, missing, and filled teeth (DMFT) index (21), where “D” (decayed) denotes the present number of teeth with untreated and active caries, “M” (missing) expresses how many teeth are missing and have been extracted due to caries, and “F” (filled) indicates how many teeth have been treated due to caries lesions. Radiography was not applied. The maximum number of teeth was 28 (excluding “wisdom teeth”). A DMFT score of 28 denotes a mouth where all teeth show caries experience (decayed, missing, or filled).

To investigate the possibility that response rates were influenced by dentition status, we conducted a short telephone interview with subjects refusing to participate in the study regarding smoking habits, education, and a self-reported evaluation of the dental health status. The refusal interview was completed by 14 irradiated cases and 45 controls. No significant differences between participants and refusals regarding smoking habits, education, self-reported caries, and periodontal status were observed.

The outcome-dependent variable was defined as the combined DMFT index. Lower values of DMFT indicate better dental health.

Irradiation status was defined as the main independent variable and was categorized dichotomously (yes/no). Gender, age at interview, education (up to 9 years, high school, academic/college degree), income compared to the National average income (much less, less, similar, more, and much more), and self-defined religiosity (secular, traditional, religious, and orthodox) were considered as other independent variables. Smoking, history of diabetes, presence of dental insurance, dental visit during the last year, and brushing teeth behavior were also investigated.

**Figure 1:**
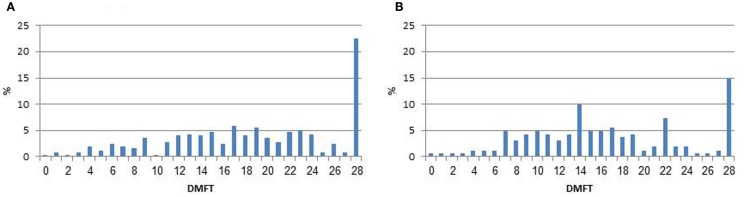
**Distribution of decayed, missing, and filled teeth (DMFT) by irradiation status**. **(A)** Irradiated subjects. **(B)** Non-irradiated comparison group.

Comparison of values of DMFT by irradiation status in the total study sample and excluding edentulous participants was performed using Wilcoxon non-parametric test. Differences of DMFT levels by study independent variables were assessed through Wilcoxon or Kruskal–Wallis tests. The DMFT levels did not distribute normally (see Figure 1), even after performing a log transformation, therefore, the distribution of DMFT among the non-irradiated was used to define quartiles. The same cut-off points were applied for the irradiated study group.

To evaluate the independent association of each study variable with dental status, an ordered univariate and multivariate logistic regression analyses were performed for the DMFT index categorized into quartiles. The proportional odds assumption for the ordered logistic regression model was checked and was found to be consistent with the data (score test *p* = 0.21).

The ORs derived from this model expressed the increase in risk per each increase in ordered category compared to the other lower categories (i.e., fourth quartile versus the combined three lower quartiles, four and three quartiles versus the combined one and two quartiles as well as two to four quartiles versus the first quartile). “Predictors with a *p*-value of 0.2 or less in the univariate analysis were included in the multivariate regression (22). Variables that did not reach statistically significance in the regression models were excluded using a backward elimination method. An additional logistic regression analysis was performed to assess the risk for higher DMFT per dose unit (using the absorbed doses to the thyroid and the salivary glands)” (16). Linear dependency of the risk on dose was assessed by introducing the term dose^2^ to the linear quadratic model; significance was tested using the Wald test.

In addition to the ordered logistic regression analyses, a quantile regression analysis using a quantile level of 0.5 (i.e., median) was performed with DMFT as a continuous variable.

## Results

A total of the 253 irradiated and 162 non-irradiated participated in the present analysis. The mean age of the study population was 58.6 (SD = 4.3). Significant statistical differences between the groups were shown for the distribution of brushing teeth habits, income, and education. Among the irradiated group, 24% complained about at least one symptom of mouth dryness (almost always or frequently), compared to 10% among the non-irradiated subjects (*p* < 0.001) (Table 1).

**Table 1:** **Demographic and dental characteristics of the study population (selected from the tinea capitis cohort and residing in two large cities in the country), by irradiation status**.

	Irradiated subjects (*n* **=** 253) (%)	Non-irradiated subjects (*n* **=** 162) (%)	*p-*value^a^
**Gender**			
Male	46.6	42.0	0.4
Female	53.4	58.0	
***Age*^b^**			
49–54	14.7	19.8	0.3
55–59	40.1	42.6	
60–64	34.5	30.9	
65–72	10.7	6.8	
**Family status^b^**			
Married	76.7	74.7	0.2
Single	2	0.6	
Separate/divorced	11.5	17.3	
Widowed	9.9	7.4	
**Education^b^**			
Primary school	42.3	22.2	0.0001
Professional/secondary school	37.9	51.2	
University/high school	19.8	26.5	
**Income (compared to the national average income)^b^**			
Higher	11.5	19.8	0.001
Similar	19	22.8	
Lower	25.7	31.5	
Much lower	41.9	22.8	
Not willing to answer/unknown	2	3.1	
**Religiosity^b^**			
Secular	9.5	13.6	0.5
Traditional	66.8	60.5	
Religious	17.8	19.8	
Orthodox	5.9	6.2	
**Ever smoking**			
Yes	50.2	48.2	0.7
No	49.8	51.9	
**Diabetes (prevalence)**			
Yes	21.3	16.7	0.2
No	78.7	83.3	
**Dental insurance^b^**			
Yes	18.3	23.5	0.2
No	81.8	76.5	
**Dentist visit last year^b^**			
Yes	61.5	63.6	0.7
No	38.5	36.4	
Dentist	56.4	58.8	0.9
Dental hygienist	32.9	37.0	0.4
**Brushing teeth^b^**			
Yes	82.9	90.1	0.04
No	17.1	9.9	
**Location of examination**			
Clinic	86.6	70.4	< 0.001
Home	13.4	29.6	
**Edentulous^b^**			
Yes	14.6	7.4	0.02
No	85.4	92.6	
**Age at first irradiation (at time of treatment)**			
<5	20.9		
5–9	53.3		
10–15	25.8		

*^a^*p*-values calculated using Chi-square test*.

*^b^At time of interview*.

Among the irradiated subjects, 37 (14.6%) demonstrated complete edentulousness (all teeth were missing) as compared to 12 (7.4%) among the non-irradiated subjects (*p* = 0.02), and 29.3 and 17.9% had no teeth in at least one jaw, respectively (*p* = 0.009). Table 2 presents data on number of healthy teeth, DMFT, D, M, and F scores by irradiation status. The mean number of caries-free teeth was 9.4 (SD = 7.5) among irradiated compared to 11.6 (SD = 7.2) among the non-irradiated subjects (*p* = 0.003). The mean DMFT level among the irradiated subjects was 18.6 (SD = 7.5) (95% CI: 17.7–19.5), compared to 16.4 (SD = 7.2) (95% CI: 15.3–17.5) among the non-irradiated subjects (*p* = 0.002). Similar results were found even when excluding edentulous (complete missing teeth) participants. As seen in Figure 1, 22.5 and 14.8% had the highest value of DMFT.

**Table 2:** **Description of dental health among study participants (selected from the tinea capitis cohort and residing in two large cities in the country), for the total group and for not edentulous subjects by irradiation status**.

	All participants			Excluding edentulous participants		
	Irradiated (*n* **=** 253)	Non-irradiated (*n* **=** 162)	*p-*value	Irradiated (*n* **=** 216)	Non-irradiated (*n* **=** 150)	*p-*value
**Healthy teeth**						
Mean (SD)	9.4 (7.5)	11.6 (7.2)	0.003	11.0 (7.0)	12.5 (6.7)	0.036
Median	9	12.5	0.002	11	13	0.02
Range	0–28	0–28		0–28	0–28	
**Decay (D)**						
Mean (SD)	0.79 (1.93)	0.64 (1.59)	0.4	0.92 (2.06)	0.69 (1.6)	0.2
Range	0–15	0–9		0–15	0–9	
**Missing (M)**						
Mean (SD)	12.1 (9.7)	8.5 (8.8)	0.0001	9.4 (7.8)	6.9 (7.1)	0.002
Range	0–28	0–28		0–27	0–26	
**Filled (F)**						
Mean (SD)	5.7 (5.4)	7.3 (5.6)	0.004	6.6 (5.2)	7.9 (5.4)	0.03
Range	0–21	0–21		0–21	0–21	
**DMFT^a^**						
Mean (SD)	18.6 (7.5)	16.4 (7.2)	0.002	17.0 (7.0)	15.5 (6.7)	0.04
Median	19	15.5	0.002	17	15	0.002
Range	0–28	0–28		0–28	0–28	
**Quartiles of DMFT**						
I (0–11)	18.2	27.8				
II (12–16)	19.4	27.2	0.005			
III (17–23)	31.6	25.9				
IV (24–28)	30.8	19.1				

*^a^DMFT – Decayed, Missing, and Filled teeth*.

As presented in Table 3, females had higher mean levels of DMFT, presenting a 47% increased risk for having high level of DMFT than males. Dental health was negatively associated with current age reaching an odd ratio of 2.4 among participants aged 65–72 years. Strong and significant inverse relationship between the DMFT index and the two socio-economic variables (education and income) was observed. Ever smoking was associated with increased DMFT (OR = 1.41 95% CI: 0.99–1.99). DMFT values were significantly lower among individuals who routinely brushed their teeth compared to those who did not (OR = 0.07 95% CI: 0.04–0.13). The mean DMFT was higher among those reported at least one symptom of mouth dryness (“almost always” or “frequently”) compared to those who did not; however, this difference did not reach statistical significance [18.3 (SD = 7.3) vs. 17.6 (SD = 7.5), *p* = 0.5, respectively].

**Table 3:** **Mean level of DMFT by independent variables and risk associated with high level of DMFT^a^ among study participants (selected from the tinea capitis cohort and residing in two large cities in the country)**.

	Irradiated			Non-irradiated			Risk adjusted for radiation status	
	*N*	Mean (SD)	*p*^b^	*N*	Mean (SD)	*p*^b^	OR^c^	95% CI
Total	253	18.6 (7.5)		162	16.4 (7.2)			
**Gender**								
Male	118	17.9 (7.9)	0.18	68	15.0 (7.5)	0.03	1.0	
Female	135	19.2 (7.2)		94	17.4 (6.9)		1.47	1.04–2.08
**Age^d^**								
49–54	37	15.0 (7.6)	0.01	32	16.3 (6.2)	0.71	1.0	
55–59	102	19.0 (7.0)		69	15.8 (6.7)		1.45	0.89–2.40
60–64	87	19.0 (7.7)		50	17.4 (8.0)		1.73	1.03–2.92
65–72	27	20.9 (7.7)		11	15.6 (9.7)		2.37	1.14–5.02
**Family status^d^**								
Married	194	18.4 (7.3)	0.46	121	16.6 (7.3)	0.45	1.0	
Single/separate/divorced	34	19.9 (7.4)		29	14.8 (6.6)		1.16	0.66–2.26
Widowed	25	18.8 (9.3)		12	18.3 (7.7)		1.22	0.71–1.89
**Education^d^**								
Primary school	107	20.3 (7.7)	0.001	36	18.4 (7.8)	0.16	1.0	
Professional/secondary school	96	18.0 (7.5)		83	16.2 (7.0)		0.58	0.39–0.88
University/high school	50	16.2 (6.5)		43	15.1 (6.8)		0.41	0.25–0.66
**Income (compared to the national average income)^d^**								
Higher	29	16.2 (6.5)	0.06	32	13.8 (6.2)	0.12	1.0	
Similar	48	17.5 (7.2)		37	16.7 (6.8)		1.55	0.87–2.76
Lower	65	18.2 (8.2)		51	16.4 (7.5)		1.57	0.91–2.72
Much lower	105	19.7 (7.5)		37	18.2 (7.7)		2.56	1.49–4.42
**Religiosity^d^**								
Secular	24	18.7 (6.9)	0.09	22	15.0 (6.1)	0.51	1.0	
Traditional	169	19.3 (7.1)		98	16.9 (7.6)		1.44	0.84–2.48
Religious/orthodox	60	16.5 (8.6)		42	15.8 (7.0)		0.9	0.49–1.65
**Ever smoking**								
No	126	17.8 (7.2)	0.08	84	15.9 (7.1)	0.38	1.0	
Yes	127	19.4 (7.8)		78	16.9 (7.4)		1.41	0.99–1.99
**Diabetes (prevalence)**								
No	199	18.5 (7.5)	0.79	135	16.2 (7.2)	0.55	1.0	
Yes	54	18.9 (7.8)		27	17.4 (7.6)		0.9	0.57–1.40
**Dental insurance^d^**								
No	207	18.8 (7.8)	0.26	124	16.9 (7.5)	0.19	1.0	0.47–1.08
Yes	46	17.8 (6.3)		38	14.8 (6.1)		0.71	
**Dental visit last year^d^**								
No	97	19.6 (8.4)	0.03	59	17.8 (7.3)	0.04	1.0	
Yes	156	18.0 (6.9)		103	15.5 (7.1)		0.61	0.42–0.88
**Brushing teeth^d^**								
No	43	25.7 (4.8)	<0.001	16	23.2 (7.1)	<0.001	1.0	
Yes	210	17.1 (7.2)		146	15.6 (6.9)		0.07	0.04–0.13

*^a^See Table 2 for the range of the DMFT quartiles*.

*^b^Differences assessed through non-parametric tests (Wilcoxon or Kruskal–Wallis)*.

*^c^Derived from an ordered logistic regression of DMFT categorized in quartiles*.

*^d^At time of interview*.

In the final ordered multivariate model, irradiation was associated with a 72% increased odds for higher DMFT level (95% CI: 1.19–2.50). Ever Smoking was associated with 53% increased odds for higher DMFT level (95% CI: 1.05–2.24). Decreased risk was found among males (OR = 0.55, 95% CI: 0.38–0.81), and among those brushing teeth regularly (OR = 0.08, 95% CI: 0.04–0.15). As the two socio-economic variables, education and income, were highly correlated, an indication of decreased risk was found among those with high level of education (OR = 0.63, 95% CI: 0.38–1.05) (Table 4).

**Table 4:** **Factors associated with high levels of DMFT^a^ – a multivariate ordered logistic regression analysis results**.

Variable	Categories	Multivariate analysis	
		OR^b^	95% CI
**A**			
Irradiation at time of treatment	Yes vs. no	1.72	1.19–2.50
Gender	Male vs. female	0.55	0.38–0.81
Age at interview	Increase of 1 year	1.04	1.0–1.09
Smoking	Ever vs. never	1.53	1.05–2.24
Brushing teeth habit at time of interview	Yes vs. no	0.08	0.04–0.15
**B1**			
Dose to the salivary gland	Increase of 1 Gy	2.21	1.40–3.50
Gender	Male vs. female	0.52	0.35–0.76
Age at interview	Increase of 1 year	1.05	1.0–1.09
Smoking	Ever vs. never	1.54	1.05–2.25
Brushing teeth habit at time of interview	Yes vs. no	0.08	0.04–0.16
**B2**			
Dose to the thyroid gland	Increase of 1 cGy	1.05	1.01–1.09
Gender	Male vs. female	0.52	0.36–0.77
Age at interview	Increase of 1 year	1.06	1.2–1.11
Smoking	Ever vs. never	1.52	1.03–2.23
Brushing teeth habit at time of interview	Yes vs. no	0.08	0.04–0.16

*A. For irradiated vs. non-irradiated and B. by dose (B1-dose to the salivary gland and B2-dose to the thyroid gland)*.

*^a^See Table 2 for the range of the DMFT quartiles*.

*^b^Derived from an ordered logistic regression of DMFT categorized in quartiles*.

A quantification of the risk by dose absorbed in the salivary gland showed an OR of 2.21 per 1 Gy (95% CI: 1.40–3.50) and OR of 1.05 per 1 cGy (centigray) (95% CI: 1.01–1.09) for dose absorbed in the thyroid gland controlling for the above confounders. The test for non-linearity based on a comparison of linear and quadratic models for the dose to the salivary gland was not statistically significant (*p* = 0.5) and to the thyroid gland (*p* = 0.7).

The results of a supplementary quantile regression analysis using a quantile level of 0.5 (i.e., median) supported the previous finding for an effect of radiation on DMFT. The irradiated group had an estimated increase of 2.35 in median DMFT (95% CI: 0.37–4.34, *p* = 0.002) controlling for gender, age, smoking, and dental hygiene compared to the non-irradiated group. For the models using doses absorbed by the salivary and the thyroid glands, the estimated increase was 3.61 (95% CI: 1.42–5.80, *p* < 0.001) for an increase of 1 Gy and 0.23 (95% CI: 0.05–0.41, *p* < 0.001) for an increase of 1 cGy, respectively. The association between age at time of irradiation and DMFT showed that while all age groups demonstrated higher odds for poor dental health, the odds reached significance only for the age groups of 5–9 and 10–15 years (OR = 1.63 95% CI: 1.05–2.51, and OR = 2.35 95% CI: 1.25–4.43, respectively) but not for the age group of 0–4 years (OR = 1.31 95% CI: 0.71–2.41) (not shown in Tables).

## Discussion

Based on a cohort that was treated in childhood with low–­moderate doses of IR to the head and neck, our results suggest that exposure to 0.2–0.4 Gy to the teeth might be associated with dental caries in adulthood. Irradiated subjects had less healthy teeth, more missing teeth, higher levels of being completely edentulous and higher DMFT scores. DMFT quartile was found to be associated with irradiation status when adjusted for the study variables. These findings were supported by a quantified analysis of dose–response using the absorbed doses to the thyroid and salivary glands as surrogates to the doses absorbed by the teeth. The consistency of the finding of statistical significance for the irradiation effect on DMFT, found in two different approaches, indicate the robustness of the results.

Those irradiated at 5–15 years of age were found to have higher odds for caries experience in adulthood than those irradiated at a younger age (0–4). It is proposed that the “window of threat” for pathogenesis, due to irradiation, is when permanent teeth are forming and erupting, at 5–15 years (23). It is interesting to note that in the assessment of the effect of IR on the development of cancer by age at exposure, an inverse association was found for most cancer types (24).

To the best of our knowledge, this is a first study that investigates the association between low-moderate dose irradiation and dental caries. Although it might be too early to speculate the precise mechanism, a plausible physiological explanation for the association could be related to an impeded salivary flow, caused by irradiation of the parotid gland. Although more irradiated than non-irradiated individuals reported symptoms of dry mouth, no direct statistical association between dryness and DMFT was demonstrated. We assume that the inability to show such association could be due to the long time span that had passed since childhood, with potential fluctuations in salivary activity over the years. Salivary secretion might have been reduced in the early years, related to caries development, but regeneration of the gland might have occurred over the life span. Moreover, as opposed to the evaluation of DMFT, dryness was estimated in this study by self-report, and no objective measurements of past and present salivary flow were performed.

Life-course psycho-social, environmental, economic, and other variables have been shown to correlate with health consequences, specifically among immigrant societies (25, 26). These factors could explain the association between IR and dental caries seen in our study. “Both study groups originated from the same cultural and socio-economic background and all experienced the potentially traumatic consequences of immigration. The exposed group underwent the additional psychological and socially distressing experience of having their hair painfully epilated by shaving and waxing before irradiation, followed by the stress and social stigma of alopecia” (16, 27). Psycho-social factors are known to potentially affect oral health preventive behavior, such as oral hygiene and dental service attendance (28). Although we have controlled for socio-economic status, we cannot discount the plausibility that the previous stressful life experience of the irradiated group could had an effect on the subsequently developing socio-economic positioning.

The general caries prevalence levels in this study, of DMFT = 16.4−18.6, are similar to global data (29, 30). The WHO has recommended that for older people (65 years and above) at least 20 functional teeth are required in order to accomplish optimal welfare (31). Therefore, a difference of two teeth with caries experience is of both statistical and clinical significance (32). A decrease in the number of teeth at older ages may indicate dental and general health deterioration. It should be noted that at an adult age, it is never completely explicit that teeth are missing only due to caries, as other factors could be present (periodontal disease, orthodontic treatment, trauma, etc.). Despite these drawbacks, it is indisputable that DMFT, including its M component, indicates dental infirmity and impeded dental health. Noteworthy is our finding that ever smoking was associated with increased odds for higher level of dental caries. A recent systematic review concluded that tobacco smoking among adults was found to be associated with increased odds of dental caries (33). It was stated that more extensive research on this topic and prospective studies are needed. The demonstrated association of smoking and dental caries in the present study support the previous information with regard to this important public health issue.

The low compliance rate that might lead to selection bias is a major concern in epidemiological studies in general as well as in this study (compliance rate of 79 and 48% among irradiated and non-irradiated individuals, respectively). It is important to note that the recruitment protocol was identical to both groups and that it included a specific explanation that the participation in the study should not be linked to poor or good dentition. This was strengthened by the non-significant differences observed between the participants and the results of the refusal questionnaire. While the possibility of a volunteer participation (due to poor dentition) could not be ruled out, we do not believe that such a bias, if it exists, will be differential between irradiated and non-irradiated.

As in all retrospective studies, recall bias due to reporting past experiences may exist in this study. In addition, not all possible confounders were considered in this study. Since the study population are Jews originated from North-Africa who share common cultural background, we might assume that both study groups have similar nutritional habits. Regarding, alcohol consumption, only five individuals reported on regular consumption. Nevertheless, detection in this study of other known risk factors for dental caries (e.g., age, gender, smoking, dental hygiene, and education) supports the validity of our results. All dental examinations were performed by one experienced dentist decreasing inter-observer variation. However, this approach could lead to another source of bias related to observer misclassification.

Although a possibility of measurement error in dosimetry in the TC cohort was found to have a minimal effect on dose–response estimation (34), the unavailability of individual doses to the teeth and use of doses absorbed by the thyroid and salivary gland as a proxy remains a limitation. Yet, the linear dose–response relationship showed in this study might indicate a genuine and causal association between irradiation and the development of dental caries.

The large irradiated cohort, the similarity of the exposed and the non-exposed groups regarding ethnic origin and immigration period, the 50 year follow-up period, and the data based on individual dental examination rather than self-reports or abstraction from medical records contribute to the advantages of this study.

Extensive research in the field of community dentistry has shown that caries prevalence is inversely proportional to socio-economic status. Our data show that the irradiated group had a much higher proportion with only primary school education, a much lower income level, and twice the percentage of subjects who did not brush their teeth. These factors could have accounted for the increase in caries in the irradiated group. However and therefore, these variables were adjusted for in the multivariate model. This analysis showed that the net effect of irradiation remained increased and significant even after controlling for the above-mentioned variables.

The shape of the dose–response curve and specifically the possible health effects of low–moderate doses of radiation are of great interest and have major implications on public health. This is specifically important in children and adolescents considering the growing exposure to diagnostic procedures, such as computed tomography (CT). The results of our study show that radiation effects on teeth are seen at much lower doses than previously thought. Opinions may differ regarding the degree to which radiation exposure increases caries risk (13, 35–37) nevertheless, our data are in line with recent publications, indicating that low dose radiations to the head, such as brain CT, may be of adverse potential (38). In line with the guidelines with the American Dental Association, these data calls for caution when using dental radiographs, mainly when performing multiple exposures and when using relatively high radiation doses (e.g., CT) (39).

In conclusion, these results add value to the understanding of the overall effects of low–moderate IR on human health.

## Author Contributions

Substantial contributions to the conception or design of the work; or the acquisition, analysis, or interpretation of data for the work (SS, AC, HS-C, JM, TA, HE-N, and YV). Drafting the work or revising it critically for important intellectual content (SS, AC, HS-C, and YV). Final approval of the version to be published (SS, AC, HS-C, JM, TA, HE-N, and YV). Agreement to be accountable for all aspects of the work in ensuring that questions related to the accuracy or integrity of any part of the work are appropriately investigated and resolved (SS, AC, and YV).

## Conflict of Interest Statement

The authors declare that the research was conducted in the absence of any commercial or financial relationships that could be construed as a potential conflict of interest.
